# Global strategies for non-communicable disease in terms of predictive, preventive and personalised medicine

**DOI:** 10.1186/1878-5085-5-S1-A4

**Published:** 2014-02-11

**Authors:** Hiroyasu Iso, Yoshihiro Kokubo

**Affiliations:** 1 Public Health, Osaka University Graduate School of Medicine, Osaka, Japan; 2 Department of Preventive Cardiology, National Cerebral and Cardiovascular Centre, Suita, Japan

## 

Human have encountered a pandemic of no-communicable disease. According to the WHO statistics [1], 36 million deaths (63% of total deaths) due to non-communicable diseases (NCDs) occurred globally in 2008. The NCDs comprise cardiovascular diseases (48%), cancers (21%), chronic respiratory disease (12%) and diabetes (3.5%). Approximately, 80% of NCD deaths occurred in low-income and middle-income countries, where a half of the deaths were premature (< 70 years of age) compared to high-income countries (a quarter). NCDs accounted for two-thirds of all years lived with disability in low-income and middle-income countries.

The WHO global action plan for the prevention and control of NCDs for 2015 to 2025 is to integrate available preventive and curative interventions through a whole government, whole society and health-in-all policies approach in order to reduce their morbidity, disability and premature mortality [2]. The action plan comprises three main pillars, i.e. surveillance, prevention and health care, which correspond to predictive, preventive and personalised medicine.

All membership states of the United Nations agreed that NCDs constitute a major challenge to socioeconomic development, environmental sustainability and poverty alleviation. The nine voluntary global targets by 2025 [3] are;

1) 25% relative reduction in overall mortality from NCDs (the 25 by 25 goal)

2) 10% or more relative reduction in the harmful use of alcohol, within the national context

3) 10% relative reduction in prevalence of insufficient physical activity

4) 30% relative reduction in mean population intake of salt/sodium intake

5) 30% relative reduction in the prevalence of current tobacco use in persons aged 15+ years

6) 25% relative reduction in the prevalence of raised blood pressure or contain the prevalence of raised blood pressure according to national circumstances

7) Halt the rise in diabetes and obesity

8) 50% or more of eligible people receive drug therapy and counselling (including glycaemic control) to prevent heart attacks and strokes; Essential NCD medications and basic technologies to treat major NCDs

9) 80% availability of the affordable basic technologies and essential medicines, including genetics, required to treat major NCDs in both public and private facilities

Japan is a unique country which has succeeded in the substantial reduction of stroke and the modest reduction of ischaemic heart disease, resulting in the elongation of life expectancy since the 1960s and madding Japan as one of the countries with longest longevity [4,5].

That success has been achieved by nation-wide reduction of sodium intake, blood pressure levels, tobacco use and substantial increase in drug therapy and counselling for the control of hypertension in the prevention of strokes and heart attacks under the insurance system since 1960 [5-7]. There is, however, an emerging public health issue of the increased prevalence of hypercholesterolemia and diabetes in men and women, and overweight in men although an increase in the increase of or mortality from ischaemic heart disease has been limited [8,9]. In Japan, the high sodium intake and the high prevalence of raised blood pressure in whole, insufficient physical activity in middle-aged men, the high tobacco use in men and the increased (although low) prevalence of diabetes in men and women are targets for the implantation of global action plan.

Each county has its own emphasis on the targets depending on lifestyles, risk factor levels and profiles of NCDs. The WHO global action plan stimulates the membership states of the United Nations to implement cost-effective surveillance, preventive and personalised interventions to aim for the achievement of the global goal.
